# Supplementary figures and images for: Cigarette smoking and reasons for leaving school among school dropouts in South Africa
Source: BMC Public Health. 2019 Jan 30;19:130. doi: 10.1186/s12889-019-6454-5 (PMC6354377; doi:10.1186/s12889-019-6454-5)

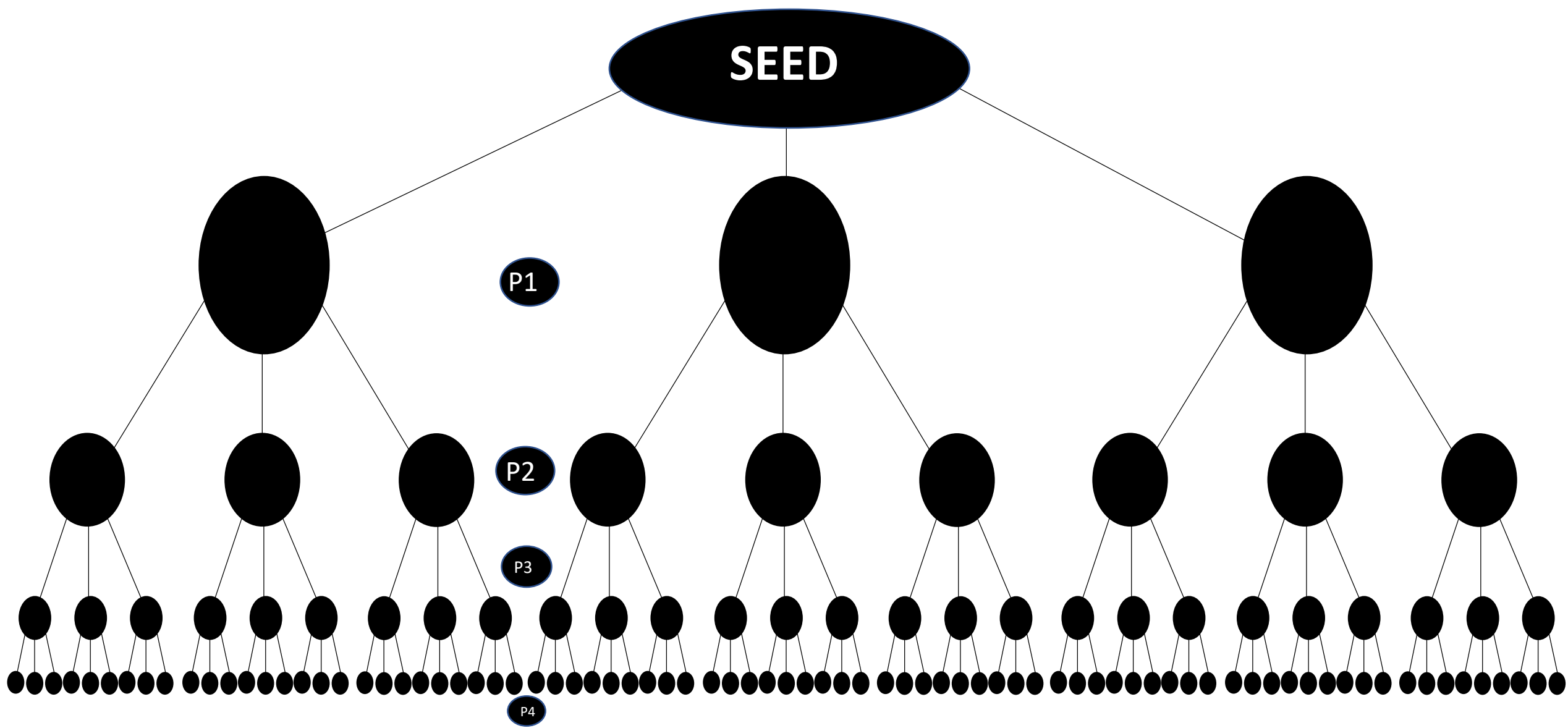

Supplement: Supplementary file 1 — Respondent Driven Sampling graphic. The file shows the full graphic representation of all phases in the respondent driven sampling used in this study (PDF 367 kb) [file 12889_2019_6454_MOESM1_ESM.pdf]
