# Supplementary material for: Cigarette smoking and reasons for leaving school among school dropouts in South Africa
Source: BMC Public Health. 2019 Jan 30;19:130. doi: 10.1186/s12889-019-6454-5 (PMC6354377; doi:10.1186/s12889-019-6454-5)
Supplement: Supplementary file 2 — English Questionnaire. The file contains the English questionnaire used in this study. (DOCX 75 kb) [file 12889_2019_6454_MOESM2_ESM.docx]

OUT-OF- SCHOOL YOUTH HEALTH RISK, LIFESKILLS AND SOCIO-ECONOMIC STATUS SURVEY

INTRODUCTION

This survey is being conducted by The Medical Research Council. The purpose of this survey is to obtain information about youth who are out of school regarding a number of behaviours that are related to their personal safety, violence, feelings, smoking, drinking alcohol, substance and drug use, sexual behaviour, nutrition, exercise and hygiene. A selection of young people between the ages of 13 and 20 years who are currently not attending school, in all nine provinces of South Africa, will be invited to participate in this survey.

You have received two documents. The first document is a list of questions with possible answers. The second document is the answer sheet on which you must colour in your answers. Notice that for each question there are a number of possible answers. Choose the answer that best fits what you know or do, then colour in / fill in the circle (O) corresponding to the question on the answer sheet. **This means that you will not mark your answers on the questionnaire but on the answer sheet.** If you must change an answer, erase / rub out the first answer and colour in / fill in the new answer.

Please do not write in the for “office use only” block at the top of the answer sheet. Remember to use the pencil that has been provided to you to fill in your answers**. Colour in the circle completely** and please stay within the lines. You will have to choose only **one answer for most** **questions.**

Please raise your hand if you are uncertain or need some help.

Remember that:

- this is not a test
- there is no right or wrong answer
- no parents / guardians or anyone else will see your answers
- your name will not be written down anywhere

**Remember to answer all the questions.**

**Thank you very much for participating in this survey.**

## Section A: Some information about yourself

# 1. Are you a boy (man) or a girl (woman)?

1. Boy (man)
2. Girl (woman)

**2. How old are you?**

1. 13 yrs
2. 14 yrs
3. 15 yrs
4. 16 yrs
5. 17 yrs
6. 18 yrs
7. 19 yrs
8. 20 yrs

**3. What is your race group?**

1. Black
2. Coloured
3. Indian
4. White
5. Other

**4. Are you a South African citizen?**

a. Yes

b. No

c. Don’t know

**5. What type of dwelling do you live in?**

1. A brick house
2. An apartment / flat
3. A shack
4. A hut
5. A house made of mud and stick
6. A house made of mud bricks and thatch
7. I live on the street/homeless
8. Wendy house/back dweller

**6. How many sleeping rooms are there in your house?**

1. One room
2. Two rooms
3. Three rooms
4. Four or more rooms
5. None

**7. How would you describe the area you live in?**

1. A rural area
2. An urban area
3. A peri-urban area (near a city/town)
4. I don’t know

**8. In your area, what kind of roads do you generally use (e.g. to get to shops, work etc)?**

1. I use tarred and well maintained roads
2. I use tarred and poorly maintained roads
3. I use a gravel road
4. I use both tarred and gravel roads
5. There is no road, I use the pathways

**9. How do you generally travel around in your area (e.g. to get to shops, work, etc)**

- 1. I mainly walk
  2. I use a bicycle
  3. I take a public bus or minibus taxi
  4. I go by car
  5. I use the train

**10. Which of your parents are still alive?**

a. My mother only

b. My father only

c. Both parents

d. Neither of them

e. I don’t know

**11. List all the people who live in your household (You can choose more than one answer)**

1. Mother
2. Father
3. Siblings (Brother/sister)
4. Aunt/uncle
5. Grandfather
6. Grandmother
7. Boyfriend/girlfriend
8. Own child/children

**12. The last time you were in school, you were in:**

1. Grade 7 or lower
2. Grade 8
3. Grade 9
4. Grade 10
5. Grade 11
6. Grade12

**13. The last year you were in school, was in?**

a. 2005 or earlier

b. 2006

c. 2007

d. 2008

e. 2009

f. 2010

**14. The reason you are not in school is: (You can choose more than one answer)**

1. You did not want to go back to school – no reason
2. You were pregnant or made someone pregnant
3. You needed to work to help your family
4. You did not have enough money to pay the school fees
5. You needed to help with looking after the house and your siblings
6. You had problems with your school work, teachers or the learners
7. The school was too far
8. Other

# 15. In the past when you attended school, how would you describe your grades in school?

1. Mostly A's (80% or more)
2. Mostly B's (70% - 79%)
3. Mostly C's (60% - 69%)
4. Mostly D's (50% - 59%)
5. Mostly E's (40% - 49%)
6. None of these grades
7. I don’t know

**16. In the past when you attended school, who paid for your school fees?**

1. My mother
2. My father
3. My brother
4. My sister
5. My Aunt or Uncle
6. I paid for myself
7. No one
8. It was free

**17. In the past when you attended school, did you work to help pay for your school fees?**

1. Yes
2. No

**18. In the past when you attended school, did you feel happy about going to school?**

1. Yes
2. No

**Please choose the response that best suits you for the following statements**

**19. My family was pleased when I left school**

a. Definitely Yes

b. Yes

c. Not sure

d. No

e. Definitely No

**20. My friends were pleased that I left school**

a. Definitely Yes

b. Yes

c. Not sure

d. No

e. Definitely No

**21. I was pleased that I left school**

a. Definitely Yes

b. Yes

c. Not sure

d. No

e. Definitely No

**22. I would like to go back to school**

a. Definitely Yes

b. Yes

c. Not sure

d. No

e. Definitely No

**Please provide some information about what you do with your time**

**(Please answer YES or NO to each statement)**

**23. I work full time**

1. Yes
2. No

**24. I go out regularly and look for work**

1. Yes
2. No

**25. I do any casual job to earn some money**

1. Yes
2. No

**26. I hang out with my friends in the game shops/malls**

1. Yes
2. No

**27. I casually walk around the neighbourhood with my friends**

1. Yes
2. No

**28. I casually walk around the neighbourhood on my own**

1. Yes
2. No

**29. I stay at home and help with house work**

1. Yes
2. No

**30. I take part in organised youth activities in the community**

1. Yes
2. No

**Please provide some information about your future plans.**

**(Please answer Yes or No to each statement)**

**31. I would like to go back to school**

1. Yes
2. No

**32. I don’t want to go back to school**

1. Yes
2. No

**33. I would like to study, but not at school**

1. Yes
2. No

**34. I would like to find a job**

1. Yes
2. No

**35. I would like to start my own business**

1. Yes
2. No

**36. I don’t know what I would like to do**

1. Yes
2. No

**37. In a normal / usual month (30 days), how much pocket money or allowance do you get?**

a. I don’t get any pocket money or allowance

b. Less than R20. 00

c. From R21.00 to R30.00

d. From R31.00 to R40.00

e. From R41.00 to R50.00

f. From R51.00 to R60.00

g. From R61.00 to R70.00

h. More than R70.00

**38.** **Some questions about your cellphone (You can choose more than one answer)**

1. I do not have a cellphone
2. I use my cellphone for Mxit only
3. I use my cellphone for SMSs only
4. I use my cellphone for calls only
5. I use my cellphone for Facebook only
6. I use my cellphone for calls, SMSs, Mxit and/or Facebook

39. Do you have a paid job? (Paid job also refers to those who are self-employed e.g. you have a shop at home)

1. Yes, I work 5 or more days a week
2. Yes, I work less than 5 days a week
3. No, I get a social grant
4. No, I am unemployed
5. No, I am ill or disabled
6. No, I am currently looking for employment
7. No, I have never looked for employment

40. Does your father (male guardian) have a paid job? (Paid job also refers to those who are self-employed e.g. your father has a shop at home)

1. Yes, works 5 or more days a week
2. Yes, works less than 5 days a week
3. No, gets a social grant
4. No, is unemployed
5. No, is ill or disabled
6. No, has retired
7. My father passed away
8. I don’t know

41. Does your mother (female guardian) have a paid job? (Paid job also refers to those who are self-employed e.g. your mother has a shop at home)

1. Yes, works 5 or more days a week
2. Yes, works less than 5 days a week
3. No, gets a social grant
4. No, is unemployed
5. No, is ill or disabled
6. No, has retired
7. My mother passed away
8. I don’t know

**42. Do you consider yourself to be disabled in any way (that is physically, mentally)?**

1. Yes
2. No

**43. Have you ever been convicted for any crime?**

a. Yes

b. No

**44. Have you ever been in prison or held in a police cell?**

1. Yes
2. No

Section B: The following questions are about your personal safety.

# 45. During the past 30 days, how often did you use a seat belt when you are in a car or other vehicle (e.g. van, taxi) driven by someone else?

1. Never (0 times)
2. Rarely (1 time)
3. Sometimes (2 or 3 times)
4. Often (4 or 5 times)
5. Very often (6 or more times)
6. Always
7. I have never been in a car or other vehicle (e.g. van, taxi) that has seat

belts

46. During the past month (30 days), how often were you in a car or other vehicle (e.g. van, taxi or bus) driven by someone who had been drinking alcohol?

1. Never (0 times)
2. Rarely (1 time)
3. Sometimes (2 or 3 times)
4. Often (4 or 5 times)
5. Very often (6 or more times)
6. I have never been in a car or other vehicle (e.g. van, taxi or bus)
7. I don’t know if the person had been drinking alcohol

47. During the past month (30 days), how often were you in a car or other vehicle (e.g. van, minibus, taxi) driven by someone who was smoking cigarettes in the car?

1. Never (0 times)
2. Rarely (1 time)
3. Sometimes (2 or 3 times)
4. Often (4 or 5 times)
5. Very often (6 or more times)
6. I have never been in a car or other vehicle (e.g. van, taxi or bus)

#

**48. Do you have a valid driver’s licence?**

1. Yes
2. No

c. I have a learner’s licence

**49. Do you drive a car or other vehicle (e.g. van) on a public road?**

1. Yes
2. No

50. How often do you use a seat belt when you yourself drive a car or other

vehicle (e.g. van, taxi or bus)?

1. Never (0 times)
2. Rarely (1 time)
3. Sometimes (2 or 3 times)
4. Often (4 or 5 times)
5. Very often (6 or more times)
6. Always
7. I do not drive a car or other vehicle (e.g. van, taxi or bus)

51. During the past month (30 days), how often did you drive a car or other

vehicle (e.g. van, taxi or bus) when you yourself had been drinking alcohol?

1. I have never driven a car or other vehicle (e.g. van, taxi or bus) when I have been drinking alcohol
2. Rarely (1 time)
3. Sometimes (2 or 3 times)
4. Often (4 or 5 times)
5. Very often (6 or more times)
6. I do not drive a car or other vehicle (e.g. van, taxi or bus)

52. During the past month (30 days), how often did you walk alongside a road

when you had been drinking alcohol?

1. I have never walked alongside a road after I had been drinking alcohol
2. Rarely (1 time)
3. Sometimes (2 or 3 times)
4. Often (4 or 5 times)
5. Very often (6 or more times)
6. I don’t drink alcohol

53. During the past month (30 days), how often did you walk alongside a road after you had been smoking dagga?

1. I have never walked alongside a road after I had been smoking dagga
2. Rarely (1 time)
3. Sometimes (2 or 3 times)
4. Often (4 or 5 times)
5. Very often (6 or more times)
6. I don’t smoke dagga

54. During the past month (30 days), how often did you walk alongside a road after you had taken other drugs?

1. I have never walked alongside a road after I had taken other drugs
2. Rarely (1 time)
3. Sometimes (2 or 3 times)
4. Often (4 or 5 times)
5. Very often (6 or more times)
6. I don’t take other drugs

**55. During your life, how often have you gambled (played any games for money)?**

1. Never (0 times)
2. Rarely (1 time)
3. Sometimes (2 or 3 times)
4. Often (4 or 5 times)
5. Very often (6 or more times)

**56. How easy or difficult will it be not to have a drink of beer, wine or other**

**alcohol drink for health reasons, when you would like to have one?**

1. Very easy
2. Easy
3. A bit difficult
4. Very difficult

**57. How easy or difficult will it be not to take drugs (dagga, heroine etc) when**

**you would like to take some?**

1. Very easy
2. Easy
3. A bit difficult
4. Very difficult

**58. How easy or difficult will it be not to have a drink of beer, wine or other alcohol**

**drink if you still need to drive home?**

a. Very easy

b. Easy

c. A bit difficult

d. Very difficult

**59. How easy or difficult will it be not to gamble when you have the opportunity to do**

**so?**

1. Very easy
2. Easy
3. A bit difficult
4. Very difficult

Section C: The following questions are about violence-related behaviour.

60. During the past month (30 days), how often did you carry a weapon such as a gun, knife, panga or kierrie?

1. Never (0 days)
2. Rarely (1 day)
3. Sometimes (2 or 3 days)
4. Often (4 or 5 days)
5. Very often (6 or more days)

61. During the past month (30 days), how often did you carry a gun?

1. Never (0 days)
2. Rarely (1 day)
3. Sometimes (2 or 3 days)
4. Often (4 or 5 days)
5. Very often (6 or more days)

62. During the past month (30 days), how often did you carry a knife?

1. Never (0 days)
2. Rarely (1 day)
3. Sometimes (2 or 3 days)
4. Often (4 or 5 days)
5. Very often (6 or more days)

63. During the past 6 months, how often has someone threatened or injured you with a weapon such as a gun, knife, panga, or kierrie in your community (at home, work or streets)?

1. Never (0 times)
2. Rarely (1 time)
3. Sometimes (2 or 3 times)
4. Often (4 or 5 times)
5. Very often (6 or more times)

64. During the past 6 months, how often have you threatened or injured someone with a weapon such as a gun, knife, panga, or kierrie in your community (at home, work or streets)?

1. Never (0 times)
2. Rarely (1 time)
3. Sometimes (2 or 3 times)
4. Often (4 or 5 times)
5. Very often (6 or more times)

# 65. During the past month (30 days), what was the one way in which you were bullied the most? (Select only one response)

- 1. I was never bullied
  2. I was called mean names, was made fun of, or was teased in a hurtful way
  3. I was hit, kicked, pushed, shoved around or locked indoors
  4. Others told lies or spread false rumours about me and tried to make people dislike me
  5. I was made fun of because of my race or colour
  6. I was made fun of because of my religion
  7. I was bullied because of my weight (underweight or overweight)
  8. I was bullied in some other way

66. During the past 6 months, how often were you in a physical fight (e.g. punching, hitting)?

1. Never (0 times)
2. Rarely (1 time)
3. Sometimes (2 or 3 times)
4. Often (4 or 5 times)
5. Very often (6 or more times)

67. During the past 6 months, how often were you in a physical fight (e.g. punching,

hitting) in which you were injured and had to be treated by a doctor or nurse?

1. Never (0 times)
2. Rarely (1 time)
3. Sometimes (2 or 3 times)
4. Often (4 or 5 times)
5. Very often (6 or more times)

68. During the past 6 months, how often were you in a physical fight (e.g. punching, hitting) in your community (home, work or streets)?

1. Never (0 times)
2. Rarely (1 time)
3. Sometimes (2 or 3 times)
4. Often (4 or 5 times)
5. Very often (6 or more times)

69. During the past 6 months, how often have you watched a physical fight (e.g. punching, hitting) in your community (home, work or streets)?

1. Never (0 times)
2. Rarely (1 time)
3. Sometimes (2 or 3 times)
4. Often (4 or 5 times)
5. Very often (6 or more times)

70. During the past 6 months, how often did you attempt to stop a physical fight (e.g. punching, hitting) in your community (home, work or streets)?

1. Never (0 times)
2. Rarely (1 time)
3. Sometimes (2 or 3 times)
4. Often (4 or 5 times)
5. Very often (6 or more times)

**71. During the past 6 months, whenever there was a physical fight in your community (home, work, streets), you mostly:**

1. Walked away
2. Called for help for eg. from the police
3. Tried to separate the fight
4. Stood and watched
5. Stood and encouraged the fight
6. Used your cellphone to video the fight
7. There was no physical fight in my community

# 72. During the past 6 months, have you been a member of a gang?

1. Yes
2. No

**73. During the past 6 months, have you been approached to join a gang?**

1. I have never been approached to join a gang
2. I have been approached to join a gang but refused
3. I have been approached to join a gang and was forced into joining the gang
4. I have been approached to join a gang and am now a member of a gang
5. I have joined a gang to earn money

74. During the past 6 months, did your boyfriend or girlfriend ever hit, smack (slap), or physically hurt you on purpose?

1. Yes
2. No
3. I do not have a boyfriend or girlfriend

75. During the past 6 months, did you ever hit, smack (slap), or physically hurt your boyfriend or girlfriend on purpose?

1. Yes
2. No
3. I do not have a boyfriend or a girlfriend

**76. Have you ever been physically forced to have sex (when the penis enters the vagina or anus) when you did not want to?**

1. Yes
2. No

# 77. Have you ever physically forced someone to have sex (when the penis enters the

# vagina or anus) when he/she did not want to?

1. Yes
2. No

Section D: The following questions are about sad feelings and attempts at suicide. Sometimes people feel so depressed about the future that they may consider attempting suicide that is, taking some action to end their own life.

**78. During the past 6 months, have you ever felt so sad or hopeless that you stopped doing some usual activities for two weeks or more in a row?**

1. Yes
2. No
3. I don’t know

**79. During the past 6 months, have you ever felt so sad or hopeless that you needed to**

**seek treatment from a doctor, counsellor or clinic?**

1. Yes
2. No

80. During the past 6 months, did you ever seriously consider attempting suicide (that is take some action to end your life)?

1. Yes
2. No

81. During the past 6 months, did you make a plan about how you would attempt suicide (that is take some action to end your life)?

1. Yes
2. No
3. I have never thought of attempting suicide

82. During the past 6 months, how many times did you actually attempt suicide (that is take some action to end your life)?

1. 0 times
2. 1 time
3. 2 or 3 times
4. 4 or 5 times
5. 6 or more times

**83. If you attempted suicide during the past 6 months, did any attempt result in an**

**injury, poisoning, or overdose that had to be treated by a doctor or nurse?**

1. I did not attempt suicide (that is take some action to end my

life) during the past 6 months

1. Yes
2. No

Section E: The following questions are about smoking.

**84. During the past month (30 days), on how many days did you smoke cigarettes?**

1. 0 days
2. 1 or 2 days
3. 3 to 5 days
4. 6 to 9 days
5. 10 to 19 days
6. 20 to 29 days
7. All 30 days

**85. During the past month (30 days), have you ever smoked any form of tobacco**

**products other than cigarettes (e.g. cigars, little cigars, pipe)?**

1. Yes
2. No

# 86. During the past month (30 days), have you ever used any form of smokeless tobacco products (e.g. chewing tobacco, snuff)?

1. Yes
2. No

87. How old were you when you first tried a cigarette?

1. I have never smoked cigarettes
2. 7 years old or younger
3. 8 or 9 years old
4. 10 or 11 years old
5. 12 or 13 years old
6. 14 or 15 years old
7. 16 years old
8. 17 years or older

88. During the past year, have you ever tried to stop smoking?

1. I have never smoked
2. I did not smoke during the past year
3. Yes, I tried to stop smoking
4. No, I did not try to stop smoking

**89. During the past week (7 days), on how many days have people smoked in your presence?**

1. 0 days
2. 1 to 2 days
3. 3 to 4 days
4. 5 to 6 days
5. 7 days

90. Do your parents / guardians smoke?

1. Both my parents / guardians **do not** smoke
2. Both my parents / guardians smoke
3. Only my father / male guardian smokes
4. Only my mother / female guardian smokes
5. I don’t know

**91. When I am in the company of friends who smoke, I usually:**

- 1. Walk away when they start smoking
  2. Refuse to smoke when they offer me
  3. Refuse and try to get them to stop smoking
  4. Make an excuse for not wanting to smoke with them
  5. Pretend that I’ll join them later then leave
  6. Join them and smoke

**92. When I am with my friends, they usually:**

a. Discourage me from smoking

b. Encourage me to smoke

c. Don’t care whether I smoke or not

**93. When I am with my parents, they usually:**

a. Discourage me from smoking

b. Encourage me to smoke

c. Don’t care whether I smoke or not

**94. How easy or difficult will it be not to smoke for health reasons, when you**

**would like to have a smoke?**

1. Very easy
2. Easy
3. A bit difficult
4. Very difficult

**95.** **How easy or difficult will it be to refuse a cigarette, when you do not want to**

**smoke, and your best friend offers you?**

1. Very easy
2. Easy
3. A bit difficult
4. Very difficult

Section F: The following questions are about drinking alcohol. This includes drinking beer, cider, wine, home brew and spirits such as brandy, gin, vodka, or whiskey. For these questions, drinking alcohol does not include drinking a few sips of traditional beer or wine for religious purposes.

96. During your life, how often have you had at least one drink of alcohol (e.g. a beer, a glass of wine, or a ‘tot’ of brandy)?

1. Never (0 days)
2. Rarely (1 or 2 days)
3. Sometimes (3 to 9 days)
4. Often (10 to 19 days)
5. Very often (20 or more days)

97. How old were you when you had your first drink of alcohol (e.g. a beer, a glass of wine, or a ‘tot’ of brandy) other than a few sips?

1. I have never had a drink of alcohol
2. I have only had a few sips of alcohol
3. 8 years old or younger
4. 9 - 10 years old
5. 11 - 12 years old
6. 13 - 14 years old
7. 15 - 17 years old
8. 18 years old or older

98. During the past month (30 days), how often did you have at least one drink of alcohol (e.g. a beer, a glass of wine, or a ‘tot’ of brandy)?

1. Never (0 days)
2. Rarely (1 to 5 days)
3. Sometimes (6 to 9 days)
4. Often (10 to 19 days)
5. Very often (20 to 30 days)

99. During the past month (30 days), how often did you have 5 or more drinks of alcohol (e.g. a beer, a glass of wine, or a ‘tot’ of brandy) in a row, that is, within a couple of hours?

1. Never (0 days)
2. Rarely (1 to 5 days)
3. Sometimes (6 to 9 days)
4. Often (10 to 19 days)
5. Very often (20 to 30 days)

**100. You are at a party and some of your friends are drinking beer. You do not want to drink so you:**

1. Say no: No thank you
2. Say it like it is: No thanks, I do not drink
3. Make an excuse: No thanks, I am in a hurry now, I have to leave
4. Wait a bit: No thanks. Maybe later
5. Change the topic: Say no and talk about something else.

**101. When I am with my friends, they usually:**

a. Discourage me from drinking alcohol

b. Encourage me to drink alcohol

c. Don’t care whether I drink alcohol or not

**102. When I am with my parents, they usually:**

a. Discourage me from drinking alcohol

b. Encourage me to drink alcohol

c. Don’t care whether I drink alcohol or not

Section G: The following questions are about dagga / hashish (marijuana) use. Dagga / hashish (marijuana) is also called zol or ganja.

103. During your life, how often have you used dagga / hashish (marijuana)?

1. Never (0 times)
2. Rarely (1 or 2 times)
3. Sometimes (3 to 9 times)
4. Often (10 to 19 times)
5. Very often (20 or more times)

104. How old were you when you tried dagga / hashish (marijuana) for the first time?

1. I have never tried dagga / hashish (marijuana)
2. 8 years old or younger
3. 9 or 10 years old
4. 11 or 12 years old
5. 13 or 14 years old
6. 15 or 16 years old
7. 17 years old or older

**105. During the past month (30 days), how often did you use dagga /hashish** (**marijuana)?**

1. Never (0 days)
2. Rarely (1 to 5 days)
3. Sometimes (6 to 9 days)
4. Often (10 to 19 days)
5. Very often (20 to 30 days)

**106. If I want to smoke dagga it will be:**

- 1. Very difficult for me to get
  2. It will be easy for me to get
  3. I know contacts in my community who can get dagga
  4. I do not have the money for dagga
  5. I do not want to take dagga ever

**107. When I am with my friends, they usually:**

a. Discourage me from using dagga

b. Encourage me to use dagga

c. Don’t care whether I use dagga or not

**108. When I am with my parents, they usually:**

a. Discourage me from using dagga

b. Encourage me to use dagga

c. Don’t care whether I use dagga or not

Section H: The following questions are about drug use.

**109. During your life have you ever taken any drug like heroin, mandrax, sugars or tik?**

1. Yes
2. No

110. During your life, how often have you sniffed glue, breathed the contents of aerosol spray cans, or inhaled any paint thinners, petrol or benzine to get high?

1. Never (0 times)
2. Rarely (1 or 2 times)
3. Sometimes (3 to 9 times)
4. Often (10 to 19 times)
5. Very Often (20 or more times)
6. I don’t know this drug

**111. During your life, how often have you taken a drug called TIK (also known as crystals, globes, speed, lolly, popeyes or straw)?**

1. Never (0 times)
2. Rarely (1 or 2 times)
3. Sometimes (3 to 9 times)
4. Often (10 to 19 times)
5. Very Often (20 or more times)
6. I don’t know this drug

**112. During your life, how often have you used mandrax (also called buttons, white pipe, indanda)?**

1. Never (0 times)
2. Rarely (1 or 2 times)
3. Sometimes (3 to 9 times)
4. Often (10 to 19 times)
5. Very Often (20 or more times)
6. I don’t know this drug

113. During your life, how often have you used any form of cocaine, including powder, crack, or rocks?

1. Never (0 times)
2. Rarely (1 or 2 times)
3. Sometimes (3 to 9 times)
4. Often (10 to 19 times)
5. Very Often (20 or more times)
6. I don’t know this drug

114. During your life, how often have you used heroin?

1. Never (0 times)
2. Rarely (1 or 2 times)
3. Sometimes (3 to 9 times)
4. Often (10 to 19 times)
5. Very Often (20 or more times)
6. I don’t know this drug

115. During your life, how often have you used a needle to inject any illegal drug into your body?

1. Never (0 times)
2. Rarely (1 or 2 times)
3. Sometimes (3 to 9 times)
4. Often (10 to 19 times)
5. Very Often (20 or more times)

116. During your life, how often have you used any other illegal drugs not mentioned

above such as ecstacy, LSD, magic mushrooms?

1. Never (0 times)
2. Rarely (1 or 2 times)
3. Sometimes (3 to 9 times)
4. Often (10 to 19 times)
5. Very Often (20 or more times)
6. I don’t know these drugs

**117. If I want to get any of the above illegal drugs it will be:**

a. Very difficult for me to get

1. A bit difficult for me to get
2. It will be rather easy for me to get
3. It will be very easy for me to get

**118. If I want to buy any of the above illegal drugs it will be:**

1. Very difficult for me to get the money to buy
2. A bit difficult for me to get the money to buy
3. It will be rather easy for me to get the money to buy
4. It will be very easy for me to get the money to buy

119. During your life, how often have you used over-the-counter or prescription drugs

(including pain killers, cough mixtures and diet pills) to get high?

1. Never (0 times)
2. Rarely (1 or 2 times)
3. Sometimes (3 to 9 times)
4. Often (10 to 19 times)
5. Very Often (20 or more times)
6. I don’t know these drugs

120. During the past 6 months, how often has someone offered, sold, or given you an illegal drug in your community (home, work or streets)?

1. Never (0 times)
2. Rarely (1 or 2 times)
3. Sometimes (3 to 9 times)
4. Often (10 to 19 times)
5. Very Often (20 or more times)

**121. During your life, where did you get the money to buy drugs?**

1. I used my pocket money
2. I asked my parents for money and pretended it was for

something else

1. I used my own money that I worked for
2. I stole money from my family members
3. I stole items from my house and sold it
4. I did not buy drugs
5. Other

**122. Have you been involved in selling drugs to earn money?**

- 1. Yes
  2. No

**123. When I am with my friends, they usually:**

a. Discourage me from using drugs

b. Encourage me to use drugs

c. Don’t care whether I use drugs or not

**124. When I am with my parents/guardian, they usually:**

a. Discourage me from using drugs

b. Encourage me to use drugs

c. Don’t care whether I use drugs or not

**Section I: The following questions are about your sexual behaviour.**

125. Have you ever had sex (when the penis enters the vagina or anus)?

1. Yes
2. No

126. How old were you when you had sex (when the penis enters the vagina or anus) for the first time?

1. I have never had sex
2. 11 years old or younger
3. 12 or 13 years old
4. 14 or 15 years old
5. 16 years old
6. 17 years old or older

127. During your life, with how many people have you had sex (when the penis enters the vagina or anus)?

1. I have never had sex
2. 1 person
3. 2 people
4. 3 or more people

**128. During the past 3 months, with how many people did you have sex (when the penis enters the vagina or anus)?**

1. I have never had sex
2. I have had sex, but not during the past 3 months
3. 1 person
4. 2 people
5. 3 or more people

129. The last time you had sex (when the penis enters the vagina or anus), did you drink alcohol before you had sex?

1. I have never had sex
2. Yes
3. No
4. I do not remember

130. The last time you had sex (when the penis enters the vagina or anus), did you use drugs (e.g. mandrax, dagga, ecstacy) before you had sex?

1. I have never had sex
2. Yes
3. No
4. I do not remember

# 131. When you have sex (when the penis enters the vagina or anus), how often do you or your partner use a condom?

1. I have never had sex
2. We never use a condom
3. We rarely use a condom
4. We sometimes use a condom
5. We use a condom most of the time
6. We always use a condom

# 132. When you have sex (when the penis enters the vagina or anus), what one method did you or your partner mostly use to prevent pregnancy? (Select only one response.)

1. I have never had sex
2. No method was used to prevent pregnancy
3. Birth control pills
4. Condoms
5. Injection (e.g. Depo-Provera)
6. Withdrawal (penis removed from the vagina before ejaculation)
7. Morning after pill
8. Some other method

133. If you are a girl, have you ever been pregnant or if you are a boy, have you ever made someone pregnant?

1. Yes
2. No
3. I don’t know if my partner was pregnant

**134. Do you have a child / children of your own?**

1. Yes
2. No

# 135. Have you or your partner ever had an abortion?

1. Yes
2. No
3. I don’t know if my partner had an abortion
4. I/my partner has never been pregnant
5. I have never had sex

# 136. If you or your partner had an abortion, where did the abortion take place?

1. In a hospital / clinic
2. At a traditional doctor / healer
3. In another place
4. I don’t know where the abortion took place
5. I don’t know if my partner had an abortion
6. I/ my partner has never been pregnant
7. I have never had sex

# 137. Have you ever had a sexually transmitted infection (STI e.g. ‘idrop’, discharge)?

1. I have never had sex
2. I do not know what an STI is
3. No, I have never had a sexually transmitted infection (STI)
4. Yes, I have had a sexually transmitted infection (STI)
5. I don’t know

# 138. If you had a sexually transmitted infection (STI), did you have treatment?

1. I have never had sex
2. No, I did not have a sexually transmitted infection (STI)
3. Yes, I had treatment for a sexually transmitted infection (STI)
4. No, I did not have treatment for a sexually transmitted infection (STI)

# 139. Do you think that you could get the HIV infection in your lifetime?

1. Yes
2. No
3. I don’t know

# 140. Do you think that you are able to protect yourself from getting the HIV infection?

#

1. Yes
2. No
3. I don’t know

# 141. Have you ever been taught about the HIV infection and/or AIDS?

1. Yes
2. No

**142. Have you ever had an HIV / AIDS test?**

- 1. Yes
  2. No

**143. Have you been involved in sexual activity for any gain, for example money, gifts, food**

**etc. ?**

1. Yes
2. No

**Section J: The following questions are about your body weight.**

144. How would you describe your weight?

1. Very underweight (very thin)
2. Slightly underweight (thin)
3. About the right weight
4. Slightly overweight (fat)
5. Very overweight (very fat)
6. I don’t know

145. Which of the following are you trying to do about your weight?

1. Lose weight
2. Gain weight
3. Stay the same weight
4. I am not trying to do anything about my weight

146. During the past month (30 days), which one of the following did you do the

most to lose weight or to keep from gaining weight? (Select only one response)

1. Exercise
2. Eat less food, fewer calories, or foods low in fat
3. Go without eating for 24 hours or more (also called fasting)
4. Take any diet pills, powders, or liquids without a doctor’s advice
5. Vomit
6. Take laxatives
7. None of the above
8. All or some of the above

Section K: The following questions ask about food you ate or drank during the past 7 days. Think about all the meals and snacks you had from the time you got up until you went to bed. Be sure to include food you ate at home, at cafes, take-aways, restaurants, or anywhere else.

147. During the past week (7 days), how often did you eat fresh fruit?

1. Very often (6 or 7 days)
2. Often (4 or 5 days)
3. Sometimes (2 or 3 days)
4. Rarely (1 day)
5. Never (0 days)

**148. Each time you ate fresh fruit, about how many fruit did you eat?**

1. I did not eat fresh fruit
2. Less than one fruit
3. 1 fruit
4. 2 fruits
5. 3 or more fruit

149. During the past week (7 days), how often did you eat vegetables that were not cooked? (eg. lettuce, carrots, cucumber)

1. Very often (6 or 7 days)
2. Often (4 or 5 days)
3. Sometimes (2 or 3 days)
4. Rarely (1 day)
5. Never (0 days)

**150. Each time you ate uncooked vegetables (eg. Lettuce, carrots, cucumber) about how much did you eat?**

1. Less than ½ cup
2. About ½ cup
3. About 1 cup
4. About 2 cups
5. More than 2 cups
6. I did not eat uncooked vegetables

151. During the past week (7 days), how often did you eat vegetables, tinned or fresh, that were cooked? (eg. Cabbage, mixed vegetables)

- - 1. Very often (6 or 7 days)
    2. Often (4 or 5 days)
    3. Sometimes (2 or 3 days)
    4. Rarely (1 day)
    5. Never (0 days)

**152. Each time you ate cooked vegetables, about how much did you eat?**

1. Less than ½ cup
2. About ½ cup
3. About 1 cup
4. About 2 cups
5. More than 2 cups
6. I did not eat cooked vegetables

153. During the past week (7 days), how often did you drink milk or eat amasi’? (Include the milk you drank in a glass or cup, from a carton, or with cereal / porridge.)

1. Very often (6 or 7 days)
2. Often (4 or 5 days)
3. Sometimes (2 or 3 days)
4. Rarely (1 day)
5. Never (0 days)

**154. Each time you drank milk or ate “amasi”, about how many glasses did you drink or**

**eat?**

1. I did not drink milk / eat “amasi”
2. Less than 1 glass
3. 1 glass
4. 2 glasses
5. 3 or more glasses

# 155. During the past week (7 days), how often did you eat fast foods or ‘luxuries’ like a hamburger, fried chicken, boerewors roll, hotdog, hot chips, ‘gatsby’, pies, vetkoek or polony roll?

1. Very often (6 or 7 days)
2. Often (4 or 5 days)
3. Sometimes (2 or 3 days)
4. Rarely (1 day)
5. Never (0 days)

**156. Each time you ate fast foods, how big was the portion of, for example the hamburger, chicken or chips?**

1. I did not eat fast foods
2. Normal size
3. Supersize
4. Mega meal

# 157. During the past week (7 days), how often did you eat a cake, vetkoek/magwinya, biscuits, koeksisters or donuts?

1. Very often (6 or 7 days)
2. Often (4 or 5 days)
3. Sometimes (2 or 3 days)
4. Rarely (1 day)
5. Never (0 days)

**158. Each time you ate cake, vetkoek / magwinya,** **biscuits, koeksisters or donuts about how much did you eat?**

1. I did not have cake, vetkoek/magwinya, biscuits, koeksisters or donuts
2. 1 ate 1 slice of cake, 1 biscuit, koeksister or donut
3. 2 slices of cake, 2 biscuits, koeksisters or donuts
4. 3 slices of cake, 3 biscuits, koeksisters or donuts
5. 4 or more slices of cake, 4 or more biscuits, koeksisters or donuts

# 159. During the past week (7 days), how often did you drink a sweet cooldrink like Coca-cola (‘Coke’), Fanta, Pepsi or other fizzy drinks?

1. Very often (6 or 7 days)
2. Often (4 or 5 days)
3. Sometimes (2 or 3 days)
4. Rarely (1 day)
5. Never (0 days)

**160. Each time you drank a sweet cooldrink (like Coke, Pepsi, or other fizzy drinks), how many glasses did you drink?**

1. I did not drink cooldrink
2. 1 glass
3. 2 glasses
4. 3 glasses
5. 4 glasses or more

# 161. During the past week (7 days), how often did you eat meat like beef, chicken, lamb?

1. Very often (6 or 7 days)
2. Often (4 or 5 days)
3. Sometimes (2 or 3 days)
4. Rarely (1 day)
5. Never (0 days)

**162. Each time you ate meat like beef, chicken, lamb, about how much did you eat?**

1. less than ½ cup
2. ½ cup
3. 1 cup
4. 2 cups
5. more than 2 cups
6. I did not eat meat like beef, chicken or lamb

# 163. During the past week (7 days), how often did you eat maize (in any form e.g. pap, porridge, rice)?

1. Very often (6 or 7 days)
2. Often (4 or 5 days)
3. Sometimes (2 or 3 days)
4. Rarely (1 day)
5. Never (0 days)

**164. Each time you ate maize (in any form e.g. pap, porridge, rice), about how much**

**did you eat?**

1. Less than ½ cup
2. ½ cup
3. 1 cup
4. 2 cups
5. more than 2 cups

Section L: The following questions ask about physical activity.

165. In the past week (7 days), how often did you exercise or participate in physical activity for at least 20 minutes, such as soccer, netball, rugby, basketball or running?

- 1. I did not take part in physical activity in the past week (7 days)
  2. 1 day
  3. 2 days
  4. 3 days
  5. 4 days
  6. 5 days
  7. 6 days
  8. Everyday

# 166. In the past week (7 days), what was the main reason for you not to take part in physical activity? (Select only one response)

1. I took part in physical activity in the past week (7 days)
2. I did not want to take part in physical activity in the past week (7 days)
3. I was ill
4. I felt unsafe, frightened and/or scared to go out to the ground / gym to

take part in physical activity

1. I do not have the equipment / ground / gym to take part in physical

activity

1. I don’t know

**167. In the past week (7 days), how often did you participate in physical activity for at least 30 minutes, such as fast walking, slow bicycling, skating, pushing a lawn mower, mopping, polishing or sweeping the floors?**

1. I did not take part in physical activity in the past week (7 days)
2. 1 day
3. 2 days
4. 3 days
5. 4 days
6. 5 days
7. 6 days
8. Everyday

**168. On an average day, how much time do you spend watching TV, playing video games or computer games?**

1. I do not watch TV, play video games or computer games on an

average day

1. Less than 1 hour per day
2. 1 hour per day
3. 2 hours per day
4. 3 hours per day
5. 4 hours per day
6. 5 or more hours per day
7. I do not have access to a TV, video games or computer games

**Section M: The following questions ask about Hygiene and Sanitation.**

# 169. How often do you wash your hands before eating?

1. Always
2. Most of the time
3. Sometimes
4. Rarely
5. Never
6. I do not have clean water

# 170. How often do you wash your hands after going to the toilet?

1. Always
2. Most of the time
3. Sometimes
4. Rarely
5. Never
6. I do not have clean water

**171. What is the main source of drinking water and other water used by your family or household?**

- 1. Inside taps and plumbing
  2. Only one tap in the yard or standpipe for water
  3. Community or public tap or standpipe away from the house
  4. Neighbours tap or water source
  5. Water truck or tanker service
  6. River, lake, spring, pond or dam
  7. Rainwater tank
  8. Other

172. What is the main toilet and sewage facility available to you and your household /

family?

a. Inside water flush toilet

b. Outside water flush toilet

c. Outside bucket toilet

d. Septic tank toilet

e. Pit toilet/latrine

f. Using bush, fields, open spaces or rivers for toilet

If you are a girl could you please answer the following question

173. How easy or difficult is it for you to get sanitary towels (pads) when you need it?

1. Very easy
2. Easy
3. A bit difficult
4. Very difficult
5. I do not use sanitary towels (pads)

That was the last question! Thank you for filling in the questionnaire.
