# Supplementary material for: Cigarette smoking and reasons for leaving school among school dropouts in South Africa
Source: BMC Public Health. 2019 Jan 30;19:130. doi: 10.1186/s12889-019-6454-5 (PMC6354377; doi:10.1186/s12889-019-6454-5)
Supplement: Supplementary file 3 — Bivariate correlation table. The file contained the correlation table to assess associations between study measures (DOCX 102 kb) [file 12889_2019_6454_MOESM3_ESM.docx]

Cigarette Smoking and Reasons for Leaving School among School Dropouts in South Africa

Rachana Desai^1, 3^ *Liesbeth A.G. Mercken^2^ Robert A.C. Ruiter^3^ Jan Schepers^4^ Priscilla S. Reddy^1, 5^

^1^Human Sciences Research Council, Population Health, Health Systems and Innovation, Private Bag X9182, Cape Town 8000, South Africa, [Rachana.desai.rd@gmail.com](mailto:Rachana.desai.rd@gmail.com); ^2^Department of Health Promotion, CAPHRI School for Public Health and Primary Care, P.O. Box 616, 6200 MD, Maastricht University, Maastricht, The Netherlands, liesbeth.mercken@maastrichtuniversity.nl; ^3^Department of Work & Social Psychology, P.O. Box 616, 6200 MD, Maastricht University, Maastricht, The Netherlands, r.ruiter@maastrichtuniversity.nl; ^4^Department of Methodology and Statistics, P.O. Box 616, 6200 MD, Maastricht University, Maastricht, The Netherlands, jan.schepers@maastrichtuniversity.nl; ^5^Faculty of Community and Health Science, University of the Western Cape, Private Bag X17. Bellville 7535, South Africa, preddy@hsrc.ac.za.

*Corresponding author.

# Abstract

**Background:** School dropouts are at heightened risk of tobacco use compared to in-school learners. School dropouts are described as those not currently enrolled in school for the academic year, have not completed their schooling, and are between 13-20 years old. This paper examines the relationship between reasons for leaving school and past month cigarette smoking, taking into account gender differences.

**Methods:** Multiple logistic regression was used to analyse survey data (n=4185). Geographical location was also incorporated into the analysis as effect moderators. **Results:** Although no significant main effects between reasons for leaving school and tobacco use were found, results showed that those who leave school early smoke more. When examining interaction effects with gender, leaving school due to 'not being able to pay for school fees' was significantly associated with smoking, but only among girls residing in urban areas (OR= 0.327, p= .023)**.**

**Conclusions:** More research is needed to understand why learners leave school and their subsequent tobacco use. This knowledge will help researchers identify and target those students that are at risk for dropping out of school and using tobacco.

**Keywords***:* Tobacco smoking, School dropout, South Africa, Respondent driven sampling

# Background

Tobacco use remains the largest preventable cause of premature deaths, accounting for over 6 million deaths each year, worldwide [1]. In addition to the death that smoking causes, tobacco use is a risk factor for a range of disease and disability, such as lung cancer, stroke, heart disease, and chronic respiratory disease [2, 3]. According to the latest data from the WHO, the average global tobacco smoking among populations aged 15 years and older was 21% [1]. Moreover, South Africans aged 15 years and older reported past month tobacco smoking as high as 31.4% [4]. Globally, cigarette smoking is common among adolescents [5]. According to the Global Youth Tobacco Surveillance results, the prevalence of past month cigarette smoking among adolescents aged 13-15 years ranged from a low of 3.8% in Uganda to a high of 17.9% in Namibia [6]. In South Africa, the Global Youth Tobacco Survey (13-15 years) and Youth Risk Behavior Survey (13-20 years) reported adolescent past month cigarette smoking as high as 12.7% and 17.6% respectively [7, 8]. Adolescents are also more likely to initiate cigarette use between the ages of 12-14 years [7-9]. Therefore, it appears that adolescents in South Africa are at heightened risk for tobacco use.

Most tobacco smoking studies in South Africa have focused on adolescents attending school. Those who have never enrolled in school or students leaving before attaining their high school diploma are often overlooked [10]. Globally, data at the end of the 2013 school year showed 124 million children and adolescents either never started school or dropped out, with nearly half living in sub-Saharan Africa [11]. In South Africa, an estimated 4% dropped out of primary school (age 13 years and below) and 12% dropped out in high school (from age 15 years old) at the end of the 2014 school year [12, 13]. The literature suggests that school dropouts reported cigarette smoking as high as 58% in the U.S and 22.6% in a small South African urban area [10, 14, 15]. School dropouts are more likely to take up tobacco smoking, as they are not guided by school-based interventions and the supervision and mentoring of teachers and peers [10, 16-18]. Therefore, school dropouts may be more vulnerable to developing tobacco-related diseases and disability than their school-going counterparts.

Reasons to stay out of school are often complex and multifaceted [19]. A number of studies conducted in high-income countries identified various reasons related to school dropout such as low academic performance [20-24], single-headed families [20-24], low socioeconomic status [10, 23, 24], and substance use and abuse [10]. In South Africa, reasons for dropping out of school have also been attributed to boredom [14, 25], bullying [21], illness [26], community violence [23] family support (pregnancy, getting someone pregnant or seeking employment to support the family) [14, 23], and school-related issues (disciplinary consequences, poor academic performance, disliking school, and conflict with teachers) [14, 23]. These studies suggest that there are various reasons contributing to school dropout.

Drug and tobacco use among adolescents has usually been associated with school dropout, the risk of leaving school, and poor educational outcomes [10, 27-29]. Compared to in-school learners, school dropouts reported significantly higher rates of cigarette smoking [14, 21]. To our knowledge, only two studies have investigated the relationship between reasons for leaving school and risky behaviour, namely crime and substance use [30-32]. These studies found that those who leave school to be with their friends, or dropout due to poor school performance, were more likely to engage in substance use, smoking and delinquency than those who leave school for family-related reasons [30-32]. Previous studies have focused on substance use in general, encompassing the use of tobacco, inhalants, hallucinogens, and alcohol. There has been limited focus on understanding the relationship between the various reasons for leaving school and cigarette smoking specifically. Understanding these differences can inform programme developers to formulate differential cessation programmes for school dropouts or those at risk for dropping out.

Gender differences may be found when examining the relationship between reasons for leaving school and cigarette smoking. Studies have shown that boys smoke more than girls, globally as well as in South Africa [1, 33]. Reasons for leaving school are also known to vary across gender. A review of the literature concluded that boys often drop out of school due to disciplinary problems, low academic achievement [34], or to seek employment to contribute towards the family income [14, 22]. Girls are more likely to leave school due to pregnancy and caretaking responsibilities [14, 22]. A South African study reported that girls were more likely to drop out of school due to bullying [21]. Therefore, based on the literature, we also expect gender differences in the relationship between reasons for leaving school and cigarette smoking.

The goal of this study was to investigate the association between various reasons for leaving school and cigarette smoking, taking into account possible gender differences. The knowledge gained in this study can contribute towards understanding the profile of school dropouts at risk for tobacco use in South Africa.

## Methods

## Study Design

Data collection took place between 2010 and 2011 and followed a cross-sectional design. Four of the nine provinces (Kwazulu Natal, Western Cape, Mpumalanga, and Gauteng) in South Africa were selected using non-probability sampling. The various language and racial groups (black African, White, Indian, Coloured, Other) of South Africa are represented by these provinces. In this study, participants were school dropouts who met the criteria of not currently being enrolled in school for the entire academic year, have not completed their schooling, and are between 13-20 years old. School dropouts are considered to be a “hidden population” with no existing register or national database for locating them. Therefore, respondent driven sampling (RDS) was an appropriate method for recruiting school dropouts [35].

A stratified cluster sample design was used to select schools (n=85) as a starting point for recruiting the initial school dropouts or “seeds.” Lists of school dropouts from the schools were obtained. Those on the list who met the criteria were contacted and formed the initial seeds. The goal was twenty “seeds” per school site. If schools were unable to provide lists of school dropouts, survey administrators recruited seeds directly from the community, such as approaching young people in the community who appeared to meet the initial criteria.

Each seed was required to identify up to three school dropouts to participate in the study. These participants formed the “first phase” of sampling and were themselves asked to identify and refer a further three school dropouts, and so on. Up to four phases of recruitment were conducted (Figure 1) (four phases of recruitment depicted in Additional file 1) [36]. A coupon system was used to keep track of the RDS recruitment chain. Each respondent received three coupons and invitation cards to recruit three other school dropouts to participate in the survey. The coupons were designed to tear off so the recruiter could keep the left half of the coupon, and the potential recruit the right half. The potential recruit was required to arrive at the survey site with their half of the coupon to complete a survey if interested. As proof of recruitment, the recruiter also returned to the survey site (in a local community hall or school) with their half of the coupon to collect monetary incentives for each participant they successfully recruited into the survey [37]. Each participant completed a self-administered questionnaire in one of the five languages (English, Afrikaans, isiZulu, Xhosa, and Sesotho). The questionnaire designed for this study was initially designed in English and translated into four languages, namely Afrikaans, isiZulu, Xhosa, and Sesotho (see Additional file 2). To check for consistency and correct translation, the survey was back translated from these languages to English. The self-administered questionnaire measured a range of socio-demographic characteristics and risk behaviour. All measures used in the current study are stated below.

[Insert Figure 1 here]

## Measures

### Past month cigarette smoking

Cigarette smoking in the past month was the main outcome variable. Participants were asked to pick a statement that best described their cigarette smoking patterns in the past 30 days. For the statistical analysis, the participants were then recoded as non-smokers (smoked 0 days) and smokers (smoked 1-30 days).

### Demographics

Demographic characteristics of the participants were provided by stating the province (1= Gauteng, 2= Kwazulu Natal, 3= Mpumalanga and 4= Western Cape), the area that they reside in (1= rural, 2= urban, 3= peri-urban), gender (1= boy, 2= girl), and their age. The racial categories defined by the Department of Labour were used to classify participant's race (1= black African, 2= Coloured, 3= Indian, 4= White, 5= Other). Racial categories allow investigation of ongoing health disparities that have endured post-Apartheid and were not used with the intention of reifying social constructions developed during the Apartheid era [38].

*The timing of the dropout*

Participants were asked to indicate the last grade they were in before leaving school (grade 7 – 12).

### Reasons for leaving school

Eight items were used to measure reasons for leaving school (0 = No, 1= Yes). Seven items represented each a different specific reason to leave school (i.e., no reason for leaving school, being pregnant or made someone pregnant, not enough money to pay school fees, working to support the family, had to help with looking after the house and siblings, the school was too far, and difficulties with school work, teachers or the learners) and one item represented other reasons not mentioned. Participants were allowed to select more than one reason. Each reason was treated as a dichotomy in the analysis.

## Analysis

Statistical analysis was conducted using IBM SPSS version 24. Descriptive statistics were used to describe the sample. Gender was cross-tabulated against study measures. A Spearman's correlation analysis was used to assess the association between study measures. The strengths for the Spearman's correlation were classified as weak (.1 ≤ r ≤ .3), moderate (.3 ≤ r ≤ .5), or strong (r ≥ .5) [39]. The prevalence past month tobacco use was examined against demographic variables, reasons for leaving school, and timing of the dropout. A Chi-square analysis of equal proportions was used to determine significant differences between categories. A pairwise check of overlapping confidence intervals was conducted to determine significant differences within categories. Logistic regression analysis was used to investigate the association between reasons for leaving school, covariates, and cigarette smoking. Moreover, the moderating effect of gender was examined in the model^a^. In the case of significant interactions, simple effects analyses were conducted to further examine the nature of the interaction [40]. All estimates were considered to be statistically significant at the 5% level of significance (p < .05).

# Results

# *Socio-demographic profile of the participants*

Of the total 4432 respondents who completed the survey, 137 respondents did not answer the tobacco smoking question and a further 110 respondents did not indicate a reason for leaving school. Therefore the final sample was 4185. As seen in Table 1, respondents most common reasons for dropping out of school were: no reason for leaving (boys= 20.8%, girls= 18.9%), they were pregnant or made someone pregnant (boys= 17.8%, girls= 19.8%), and they did not have enough money to pay school fees (boys= 18.1%, girls= 18.8%). More than half (58%) were boys and the majority classified themselves as black African (72.5%). The mean age was 17.4 years (SD= 1.6) and 20% had left school in grade 10 (age 16 onwards). Less than half (46.1%) resided in rural areas and 27.7 % resided in the Western Cape. In addition, bivariate correlation analysis was used to assess associations between study measures (see Additional File 3). At the p= .05 level of significance, the correlation coefficients were mostly weak and non-significant.

[Insert Table 1 here]

## Prevalence of past month tobacco smoking

Overall, the prevalence of past month tobacco smoking among school dropouts was 50.2%. As shown in Table 2, boys (61.6%, [95% CI: 59.6-63.5]) had a significantly higher prevalence of past month cigarette smoking than girls (33.9%, [95% CI: 31.6-36.2]). Those residing in Western Cape (69.5%, [95% CI: 66.7-72.1]) significantly smoked more than those living outside the Western Cape. Participants living in urban areas (56.8%, [95% CI: 53.9-59.8]) also smoked more than those in rural areas (44.4%, [95% CI: 42-46.8]). The prevalence of tobacco smoking was high among those who left school in grade eight (56.8%, [95% CI: 53-60.4]) and grade nine (58.2%, [95% CI: 54.5-61.9]) compared to those leaving school later (Table 2).

[Insert Table 2 here]

## Development of the logistic regression model

The relationship between past month smoking and reasons for leaving school, moderated by gender was investigated. Covariates that were significantly associated with the smoking variable were included in the model. Further, it was found that the gender x reasons for leaving school interaction terms were non-significant (p's> .05). Since the various provinces and areas showed significant differences on the smoking variable, these variables were included in a four-way interaction model: gender x reasons for leaving x province x area. The model was reduced by removing higher order terms based on non-significant omnibus tests, followed by eliminating lower order non-significant terms. In line with our original hypotheses, the terms reasons for leaving school and reasons for leaving x gender were kept in the models, irrespective of their significance.

## Reasons for leaving school and cigarette smoking

The final model shown in Table 3, revealed a significant three-way interaction of gender x not having enough money to pay for school fees x area. Simple effects analysis, shown in Table 4, revealed a significant two-way interaction of gender with "not enough money to pay for school fees" in urban areas as opposed to rural and peri-urban areas (OR=0.297, p=.016, [95% CI: 0.110-0.800]). To investigate this significant two-way interaction in depth, separate analysis for boys and girls were performed. Results showed that leaving school due to not having enough money to pay for school fees was associated with less cigarette smoking, but only among girls living in urban areas (OR=0.327, p=.023, [95% CI: 0.158-0.872] ). The final model, as shown in Table 3, further implied the following significant two-way interactions: The effect of being pregnant or made someone pregnant in urban areas (OR=0.542, p= .011, [95% CI: 0.338-0.867]) is different compared to that effect in rural areas (OR = 1.810,[ 95% CI: 0.614-5.336]). The effect of “other” reasons for leaving in Mpumalanga (OR=3.761) is different (p=.00, [95% CI: 1.858-7.616]) from that effect in Gauteng (OR=0.82, [95% CI: 0.252-2.671]). Further simple effects analysis revealed non-significant effects.^b^

[Insert Table 3 here]

Table 4 Simple effects analysis for significant interaction effects in the model with gender as a moderator

| Gender * Not enough money to pay for school fees | B | S.E | Wald | p-value | Odds ratio | 95% CI |
| --- | --- | --- | --- | --- | --- | --- |
| Simple effects in different areas |  |  |  |  |  |  |
| Urban | -1.214 | .506 | 5.769 | .016 | 0.297 | [0.110 – 0.800] |
| Girls | -0.990 | .435 | 5.172 | .023 | 0.327 | [0.158 – 0.872] |
| Boys | 0.193 | .270 | 0.511 | .476 | 1.213 | [0.715 – 2.057] |

# Discussion

The results of this study confirm that cigarette smoking was common among school dropouts in this sample. Past month cigarette smoking was reported by 50.4% of the respondents with boys smoking twice as much compared to girls. Earlier studies also confirm that school dropouts exceeded the rate of cigarette smoking compared to in-school learners who reported 17.6% and 13.6% according to two national studies [7, 8]. In comparison to in-school learners who reported 25% smoking in the Western Cape province, cigarette smoking among school dropouts is as high as 69.5% in the Western Cape and 56.8% in the urban areas. Those leaving school in grade 8 and 9 appeared to smoke more than those leaving school later. In contrast, in-school learners appear to smoke more in the later grades compared to those in grades 9 and lower [8]. These findings are worrying, particularly the fact that school dropouts are at higher risk for tobacco-related morbidity and mortality, posing a serious public health threat [10, 16-18].

This paper investigated the relationship between various reasons for leaving school and cigarette smoking. Surprisingly, no significant main effects were found between the reasons for leaving school and subsequent cigarette smoking. The few studies conducted among school dropouts have either focused on substance use in general [30] or problem behaviour [31] as a function of reasons for leaving school. Some of our findings are in line with Aloise-Younge 2002, who found that substance use did not differ among adolescents who left school due to problems with teachers or poor school performance. Aloise-Younge 2002 only found significant effects between reasons for leaving and substance use when ethnic differences were taken into account [30]. Moreover, Jarjoura (1996) found that dropping out for school-related reasons (poor grades and problems with teachers) was more strongly related to delinquency, but only among adolescents from higher income households [31].

The present study was the first study that focused solely on the relationship between reasons for leaving school and cigarette smoking. The lack of significant relationships between both concepts may be accounted for by the lack of a standardised measure used for cigarette smoking. Given that the legal age for tobacco use in South Africa is 18, participants in this study were underage and may have also underreported their cigarette smoking behaviour. Studies have furthermore shown that tobacco use in the form of waterpipe, snuff, pipes, cigars, and cigarillos are increasing in popularity among adolescents in South Africa, which were not considered in this study [41]. On the other side of the comparison, the South African literature cited reasons for leaving school such as bullying [21], boredom [25] illness [26], and community violence [23], which were also not incorporated into this study. Future studies may find it useful to consider a qualitative approach to understanding the reasons for leaving school and the impact on tobacco use among school dropouts.

The second aim of this paper was to investigate the relationship between reasons for leaving and cigarette smoking, taking into account possible gender differences. Surprisingly, no significant effects were found, only when gender differences were considered. Therefore, we examined how reasons for leaving school differed by geographical location, as well as gender. Contrary to our expectations, we found that leaving school for not having enough money to pay for school fees was associated with less cigarette smoking, but only among girls living in urban areas. A qualitative study confirm our findings and indicated that physical (poor living conditions, inability to meet school costs), social (unemployment among caregivers and single headed families) and psychological (feelings of disempowerment and despair) poverty is a contributing factor to why adolescents leave school in three poor and marginalised urban communities in South Africa [23]. This is not surprising, given that more than two out of every five youth live below the poverty line in South Africa [42]. Moreover, the HIV/AIDS pandemic has severely affected the poor communities in South Africa [43]. School expenses cannot be met due to reduced income, possibly from the illness of the highest income recipient in the household, and an increased expenditure of health services, and funerals [43, 44]. In many households affected by HIV and AIDS, girls tend to be the first to be taken out of school and the first to take on increased family responsibilities, including caring for an ailing guardian [44]. Boys may be more likely to seek employment to contribute towards the family income [22]. Consequently, boys may be able to afford purchasing cigarettes compared to girls who leave for the same reason.

The present study is not without its limitations [45]. Respondent driven sampling was conducted in four of the nine provinces of South Africa and therefore the results cannot be generalised to the entire population. However, bias that the non-random choice of seeds may have introduced is overcome through the sufficient number of phases of peer recruitment, which stabilises the composition of the sample, thereby becoming independent of the seeds from which recruitment began [37]. Data in this survey are also based on self-report and are therefore subject to the limitations of self-report bias. Although extensive literature exists on the correlates of friend and family smoking, we unfortunately did not have information on friend smoking, and a large amount of missing or unknown data was found on parent/guardian smoking. Finally, causal relationships could not be addressed due to the cross-sectional nature of the study. These limitations notwithstanding, this study provides valuable insight into the associations of cigarette smoking among school dropouts. To better elucidate causal mechanisms, future longitudinal and national studies will be needed.

# Conclusions

The present study was the first study to examine the relationship between reasons for leaving school and cigarette smoking. This study found a significant effect between reasons for leaving school and cigarette smoking when demographic factors were incorporated into the analysis, in particular, gender and geographic location. Future research should closely explore the various reasons for dropping out of school and tobacco use in South Africa not considered in this study, possibly using qualitative methods to target the correct reasons for leaving.

This knowledge will help researchers identify and target those students that are at risk for dropping out of school and tobacco smoking. Such findings will inform the recommendations made for future research efforts, as well as the development of specific policies and interventions pertaining to tobacco use among high-risk school dropouts.

**Declarations**

***Availability of data and materials***

The datasets generated and analysed during the current study are not publicly available due to participant confidentiality but are available from the corresponding author on reasonable request.

# *Ethics approval and consent to participate*

The South African Medical Research Ethics Committee granted ethical approval for the study. Permission was additionally obtained from the relevant Provincial Departments of Education and school principles to use the schools as initial points of contact. Participants, as well as the parent/guardians of participants younger than 18 years, gave written consent and assent to participate in the study.

# *Consent for publication*

# Not applicable.

# *Competing interests*

# The authors declare they have no competing interests.

# *Funding*

Funding information is not applicable

***Author contributions***

RD conceived the study, participated in its design and coordination, statistical analysis and interpretation and drafted the original and final manuscript. LM and RR supervised RD, made substantial contributions to conception and design, analysis and interpretation of data, and have been involved in revising the manuscript. JS participated in the data analysis, interpretation of the data and reviewing the manuscript. PR was the principal investigator of the study, grant holder of the project, participated in the conceptualisation, design, data collection and coordination of the project, supervised RD and contributed towards reviewing the manuscript. All authors read and approved the final manuscript.

# *Acknowledgements*

We would like to acknowledge the scholarship of the Foundation Study Fund for South African students in the Netherlands. Opinions expressed and conclusions arrived at are those of the authors and are not necessarily to be attributed to the funder. We also thank the participants, data collectors, and translators for their participation and assistance in the project.

Endnotes

^a^Similar analyses were conducted that tested the moderating effect of timing of the dropout, however, these analyses did not result in significant outcomes. ^b^Similar analyses were done for the timing of dropout but did not result in significant outcomes.

# References

1. WHO. Global health observatory (GHO) data World Health Organisation2015. <http://www.who.int/gho/en/>. Accessed 18th November 2016.

2. Reddy P, Sewpaul R. Tobacco Control and Health Human Sciences Research Council (HSRC) http://www.hsrc.ac.za/en/research-outputs/view/7016. Accessed 5th September 2016.

3. Moore S, Teixeira A, Stewart S. Effect of network social capital on the chances of smoking relapse: a two-year follow-up study of urban-dwelling adults. American Journal of Public Health. 2014;104:e72-6. doi: 10.2105/AJPH.2014.302239.

4. WHO. WHO Report on the Global Tobacco Epidemic 2011: Warning About the Dangers of Tobacco. Geneva: WHO: 2011.

5. Eriksen M, Mackay J, Ross H. The tobacco atlas: American Cancer Society; 2013.

6. Page RM, Danielson M. Multi-country, cross-national comparison of youth tobacco use: findings from global school-based health surveys. Addictive Behaviors. 2011;36:470-8.

7. Reddy SP, James S, Sewpaul R, Koopman F, Sifunda S, Masuka P, et al. The 2008 Global Youth Tobacco Survey: The 3rd GYTS in South Africa. Cape Town: South African Medical Research Council;2010.

8. Reddy SP, James S, Sewpaul R, Sifunda S, Ellahebokus A, Kambaran NS, et al. Umthente Uhlaba Usamila: The 3rd South African national youth risk behaviour survey 2011. Cape Town: South African Medical Research Council; 2013.

9. Townsend L, Flisher AJ, Gilreath T, King G. A systematic review of tobacco use among sub‐Saharan African youth. Journal of Substance Use. 2006;11:245-69.

10. Townsend L, Flisher AJ, King G. A systematic review of the relationship between high school dropout and substance use. Clinical Child and Family Psychology Review. 2007;10:295-317.

11. UNESCO. A growing number of children and adolescents are out of school as aid fails to meet the mark, Policy Paper 22, Fact Sheet 31. Paris and Montreal: EFA Global Monitoring Report and UNESCO Institute for Statistics; 2015.

12. Department of Basic Education. Report on dropout and learner retention strategy to portfolio committee on education 2011. <http://www.education.gov.za/Portals/0/Documents/Reports/REPORT%20ON%20DROPOUT%20AND%20ETENTION%20TO%20PORTFOLIO%20COMMITTEE%20JUNE%202011.pdf?ver=2015-03-20-120521-617>. Accessed 3rd March 2017.

13. FHI360. National Education Profile: 2014 Update. [www.epdc.org/sites/default/files/documents/EPDC%20NEP_South%20Africa.pdf](http://www.epdc.org/sites/default/files/documents/EPDC%20NEP_South%20Africa.pdf). Accessed 3rd March 2017.

14. Flisher AJ, Townsend L, Chikobvu P, Lombard CF, King G. Substance use and psychosocial predictors of high school dropout in Cape Town, South Africa. Journal of Research on Adolescence. 2010;20:237-55.

15. Wang MQ, Fitzhugh EC, Eddy JM, Westerfield RC. School dropouts' attitudes and beliefs about smoking. Psychological Reports. 1998;82:984-6. doi: 10.2466/pr0.1998.82.3.984.

16. Flisher AJ, Chalton DO. High-school dropouts in a working-class South African community: selected characteristics and risk-taking behaviour. Journal of Adolescents. 1995;18:105-21.

17. Aloise-Young PA, Cruickshank C, Chavez EL. Cigarette smoking and perceived health in school dropouts: a comparison of Mexican American and non-Hispanic white adolescents. Journal of Pediatric Psychology. 2002;27:497-507.

18. Adebiyi AO, Faseru B, Sangowawa AO, Owoaje ET. Tobacco use amongst out of school adolescents in a Local Government Area in Nigeria. Substance Abuse Treatment, Prevention, and Policy. 2010;5:1-5.

19. Brown BA. Social hostility and the “dropout” syndrome: leadership assisting youths’ re‐entry into school? Educational Review. 2010;62:53-67.

20. Inoue K, Fukunaga T, Fujita Y, Okazaki Y. A Discussion of trends for dropping out of high School in Japan and efforts to decrease school dropouts: an examination focusing on reasons for dropping out given by dropouts. International Medical Journal. 2011;18.

21. Townsend L, Flisher AJ, Chikobvu P, Lombard C, King G. The relationship between bullying behaviours and high school dropout in Cape Town, South Africa. South African Journal of Psychology. 2008;38:21-32.

22. Stearns E, Glennie EJ. When and why dropouts leave high school. Youth & Society. 2006;38:29-57.

23. Porteus K, Clacherty G, Mdiya L, Pelo J, Matsai K, Qwabe S, et al. ‘Out of School’children in South Africa: an analysis of causes in a group of marginalised, urban 7 to 15 year olds. Support for Learning. 2000;15:8-12.

24. De Witte K, Cabus S, Thyssen G, Groot W, van den Brink HM. A critical review of the literature on school dropout. Educational Research Review. 2013;10:13-28.

25. Wegner L, Flisher AJ, Chikobvu P, Lombard C, King G. Leisure boredom and high school dropout in Cape Town, South Africa. Journal of adolescence. 2008;31:421-31.

26. Branson N, Hofmeyr C, Lam D. Progress through school and the determinants of school dropout in South Africa. Development Southern Africa. 2014;31:106-26.

27. Weybright EH, Caldwell LL, Xie HJ, Wegner L, Smith EA. Predicting secondary school dropout among South African adolescents: A survival analysis approach. South African Journal of Education. 2017;37:1-11.

28. Gasper J. Revisiting the relationship between adolescent drug use and high school dropout. Journal of Drug Issues. 2011;41:587-618.

29. Heradstveit O, Skogen JC, Hetland J, Hysing M. Alcohol and illicit drug use are important factors for school-related problems among adolescents. Frontiers in Psychology. 2017;8:1023. doi: 10.3389/fpsyg.2017.01023.

30. Aloise‐Young PA, Chavez EL. Not all school dropouts are the same: Ethnic differences in the relation between reason for leaving school and adolescent substance use. Psychology in the Schools. 2002;39:539-47.

31. Jarjoura GR. The conditional effect of social class on the dropout-delinquency relationship. Journal of Research in Crime and Delinquency. 1996;33:232-55.

32. Jarjoura GR. Does dropping out of school enhance delinquent involvement? Results from a large‐scale national probability sample. Criminology. 1993;31:149-72.

33. Reddy P, James S, Sewpaul R, Yach D, Resnicow K, Sifunda S, et al. A decade of tobacco control: The South African case of politics, health policy, health promotion and behaviour change. SAMJ: South African Medical Journal. 2013;103:835-40.

34. Doll JJ, Eslami Z, Walters L. Understanding why students drop out of high school, according to their own reports. Sage Open. 2013;3:1 - 15. doi: 10.1177/2158244013503834.

35. Wejnert C. Social network analysis with respondent-driven sampling data: a study of racial integration on campus. Social Networks. 2010;32:112-24. doi: 10.1016/j.socnet.2009.09.002.

36. Reddy SP, James S, Sewpaul R, Koopman F, Sifunda S, Masuka P, et al. Health Risk Behaviours, life skills and socio-economic status survey of Out-of-School Youth in South Africa: An investigation into sexual and other behaviours that place the next generation at risk. Cape Town: South African Medical Research Council; 2011.

37. Heckathorn DD. Respondent-driven sampling II: deriving valid population estimates from chain-referral samples of hidden populations. Social Problems. 2002;49:11-34.

38. Stats SA. Mid-year population estimates. Statistical Release. 2011;302.

39. Kotrlik JW, Williams HA, Jabor MK. Reporting and interpreting effect size in quantitative agricultural education research. Journal of Agricultural Education. 2011;52:132-42.

40. Aiken LS, West SG, Reno RR. Multiple regression: testing and interpreting interactions: Sage; 1991.

41. Shisana O, Labadarios D, Rehle T, Simbayi L, Zuma K, Dhansay A, et al. South African national health and nutrition examination survey (SANHANES-1). Cape Town: HSRC Press; 2013.

42. Stats SA. Poverty trends in South Africa: an examination of absolute poverty between 2006 and 2015. Pretoria: Statistics South Africa. 2017.

43. Collins DL, Leibbrandt M. The financial impact of HIV/AIDS on poor households in South Africa. Aids. 2007;21:S75-S81.

44. UNICEF. Girls, HIV/AIDS and education: UNICEF: New York; 2004.

45. Heckathorn DD. Respondent-driven sampling: a new approach to the study of hidden populations. Social Problems. 1997;44:174-99.

**Figure Legends**

Figure 1

Respondent Driven Sampling for Out-of-School Youth-

A Graphical Illustration of Two Sampling Phases

Additional files

Additional file 1:

Respondent Driven Sampling graphic.

The file shows the full graphic representation of all phases in the respondent driven sampling used in this study

Additional file 2:

English Questionnaire.

The file contains the English questionnaire used in this study.

Additional File 3:

Bivariate correlation table.

The file contained the correlation table to assess associations between study measures

| Table 1  Characteristics of the sample and reported reasons for leaving school per gender | | | | | | |
| --- | --- | --- | --- | --- | --- | --- |
|  | Total | | Gender | | | |
|  |  |  | Boy | Girl | | |
| Characteristics | % /Mean (SD) | n | %/Mean (SD) | n | %/Mean (SD) | n |
| Total | 100 | 4222 | 58 | 2506 | 39.7 | 1716 |
| Past month cigarette smoking |  |  |  |  |  |  |
| Smoker | 50.2 | 2056 | 61.6 | 1488 | 33.9 | 568 |
| Non – smoker | 49.8 | 2037 | 38.4 | 928 | 66.1 | 1109 |
| Reasons for leaving school |  |  |  |  |  |  |
| No reason for leaving school | 20 | 845 | 20.8 | 520 | 18.9 | 325 |
| You were pregnant or made  someone pregnant | 18.6 | 787 | 17.8 | 447 | 19.8 | 340 |
| Working to help the family | 16.8 | 708 | 17.4 | 435 | 15.9 | 273 |
| Not enough money to pay for school  fees | 18.4 | 777 | 18.1 | 484 | 18.8 | 323 |
| Had to help with looking after the  house and siblings | 5.1 | 214 | 4.9 | 123 | 5.3 | 91 |
| Problems with school work, teachers or  the learners | 10.4 | 441 | 10.7 | 267 | 10.1 | 174 |
| The school was too far | 4.4 | 185 | 4.5 | 112 | 4.3 | 73 |
| Other | 12.3 | 518 | 12.4 | 311 | 12.1 | 207 |
| Province |  |  |  |  |  |  |
| Gauteng | 23 | 971 | 26.6 | 667 | 17.7 | 304 |
| Kwazulu Natal | 27.3 | 1153 | 24.1 | 603 | 32.1 | 550 |
| Mpumalanga | 22 | 930 | 19.9 | 498 | 25.2 | 432 |
| Western Cape | 27.7 | 1168 | 29.4 | 738 | 25.1 | 430 |
| Race |  |  |  |  |  |  |
| black African | 72.5 | 2995 | 70.2 | 1716 | 75.9 | 1279 |
| Coloured | 21.8 | 899 | 23.7 | 580 | 18.9 | 319 |
| Indian | 1.7 | 70 | 2.2 | 54 | 0.9 | 16 |
| White | 1.4 | 58 | 1.2 | 29 | 1.7 | 29 |
| Other | 2.6 | 108 | 2.7 | 65 | 2.6 | 43 |
| Area |  |  |  |  |  |  |
| Rural | 46.1 | 1673 | 44.6 | 953 | 48.3 | 720 |
| Urban | 30.4 | 1103 | 32.7 | 699 | 27.1 | 404 |
| Peri-urban | 23.5 | 855 | 22.8 | 487 | 24.7 | 368 |
| Age | 17.4 (1.6) | 4215 | 17.4 (1.9) | 2458 | 17.6 (1.7) | 1683 |
| Timing of the dropout |  |  |  |  |  |  |
| Grade 7 or lower | 18.5 | 747 | 19.4 | 461 | 17.3 | 286 |
| Grade 8 | 16.8 | 677 | 17.5 | 416 | 15.8 | 261 |
| Grade 9 | 17.2 | 691 | 18.7 | 45 | 14.9 | 246 |
| Grade 10 | 20 | 805 | 19.6 | 465 | 20.6 | 340 |
| Grade 11 | 16.8 | 678 | 15.6 | 370 | 18.7 | 308 |
| Grade 12 | 10.7 | 429 | 9.2 | 219 | 12.7 | 210 |

^Standard deviation (SD)^

Table 2

Prevalence of past month tobacco smoking by demographic characteristics, the timing of drop out and reasons for leaving school

| Characteristics | Past month tobacco smoking | | | |
| --- | --- | --- | --- | --- |
|  | % | 95% confidence interval |  | n |
| Total | 50.2 |  |  | 4222 |
| Gender |  |  | p<.05 |  |
| Boy^a^ | 61.6 | [59.6-63.5] | a>b | 2416 |
| Girl^b^ | 33.9 | [31.6-36.2] |  | 1677 |
| Age |  |  | >.05 |  |
| 13 years | 48.2 | [41.4-55.2] |  | 199 |
| 14 years | 52.5 | [46.4-58.5] |  | 261 |
| 15 years | 51.5 | [45.5-57.6] |  | 260 |
| 16 years | 50.2 | [45.4-55.0] |  | 414 |
| 17 years | 54.9 | [50.5-59.1] |  | 505 |
| 18 years | 49.6 | [45.9-53.4] |  | 681 |
| 19 years | 49.5 | [47.2-51.8] |  | 1772 |
| Province |  |  | p<.05 |  |
| Gauteng ^a^ | 57.7 | [54.5-60.8] | a>b; a>c; a<d | 955 |
| Kwazulu Natal^b^ | 34.4 | [31.7-37.2] | b<d | 1157 |
| Mpumalanga^c^ | 39.5 | [36.4-42.6] | c<d | 930 |
| Western Cape^d^ | 69.5 | [66.7-72.1] |  | 1143 |
| Race |  |  | p<.05 |  |
| African^a^ | 42.8 | [41.0-44.5] | a<b; a<c; a<d | 2975 |
| Coloured^b^ | 74.6 | [71.6-77.3] | b>e | 881 |
| Indian^c^ | 65.2 | [53.3-75.5] |  | 69 |
| White^d^ | 59.3 | [46.4-71.0] |  | 59 |
| Other^e^ | 45.8 | [36.6-55.3] |  | 107 |
| Area |  |  | p<.05 |  |
| Rural^a^ | 44.4 | [42.0-46.8] | a<b | 1659 |
| Urban^b^ | 56.8 | [53.9-59.8] |  | 1089 |
| Peri – urban^c^ | 50 | [46.6-53.4] |  | 844 |
| Timing of drop out |  |  | p<.05 |  |
| Grade 7 or lower^a^ | 49.3 | [45.8-52.9] | a<b; a<c; a>f | 746 |
| Grade 8^b^ | 56.8 | [53.0-60.4] | b>d; b>e; b>f | 680 |
| Grade 9^c^ | 58.2 | [54.5-61.9] | c>d; c>e; c>f | 682 |
| Grade 10^d^ | 48.3 | [44.8-51.8] | d>f | 797 |
| Grade 11^e^ | 46 | [42.3-49.8] |  | 667 |
| Grade 12^f^ | 37 | [32.5-41.7] |  | 427 |
| Reasons for leaving school |  |  | p>.05 |  |
| No reason for leaving school | 49.9 | [46.6-53.3] |  | 843 |
| Being pregnant or made someone pregnant | 51.1 | [47.6-54.6] |  | 775 |
| Working to help the family | 53.3 | [49.6-56.9] |  | 704 |
| Not enough money to pay for school fees | 49.2 | [45.7-52.8] |  | 768 |
| Had to help with looking after the house and your  siblings | 50.9 | [44.3-57.5] |  | 216 |
| Problems with your school work, teachers or the  learners | 46.4 | [41.8-51.1] |  | 435 |
| The school was too far | 47.6 | [40.5-54.8] |  | 185 |
| Other | 52.5 | [48.1-56.8] |  | 507 |

Table 3

Logistic regression results for the model including interaction terms with province, area, and gender

|  |  |  | 95% Confidence Interval | | |  |
| --- | --- | --- | --- | --- | --- | --- |
|  | B | S.E. | Lower | Exp (B) | Upper | p-value |
| Kwazulu Natal (ref Gauteng) | -1.082* | .328 | 0.178 | .339 | 0.644 | .001 |
| Mpumalanga | 0.595 | .360 | 0.896 | 1.813 | 3.667 | .098 |
| Western Cape | -0.786* | .343 | 0.233 | .456 | 0.893 | .022 |
| Urban (ref rural) | 0.406 | .312 | 0.815 | 1.501 | 2.765 | .193 |
| Peri-urban | 0.511 | .337 | 0.861 | 1.667 | 3.229 | .130 |
| Timing of the dropout | -0.089* | .025 | 0.872 | .915 | 0.960 | .000 |
| Coloured (ref black African) | 1.020* | .127 | 2.163 | 2.772 | 3.553 | .000 |
| Indian | 0.245 | .332 | 0.667 | 1.277 | 2.447 | .461 |
| White | 0.388 | .337 | 0.761 | 1.474 | 2.856 | .250 |
| Other | 0.205 | .269 | 0.724 | 1.227 | 2.080 | .447 |
| Boys versus Girls | -0.903* | .416 | 0.179 | .405 | 0.917 | .030 |
| No reason for leaving school | -0.376 | .565 | 0.227 | .687 | 2.079 | .506 |
| Being pregnant or made someone pregnant | 0.593 | .552 | 0.614 | 1.810 | 5.336 | .282 |
| Working to help the family | 0.033 | .520 | 0.373 | 1.034 | 2.863 | .949 |
| Not enough money to pay for school fees | -0.065 | .631 | 0.272 | .937 | 3.227 | .918 |
| Had to help with looking after the house and siblings | 0.072 | .654 | 0.298 | 1.074 | 3.870 | .913 |
| Problems with your school work, teachers or the learners | 0.159 | .571 | 0.383 | 1.172 | 3.591 | .781 |
| The school was too far | -0.356 | .672 | 0.188 | .701 | 2.615 | .596 |
| Other | -0.198 | .602 | 0.252 | .820 | 2.671 | .742 |
| Gender * No reason for leaving school | 0.159 | .411 | 0.524 | 1.173 | 2.625 | .699 |
| Gender * Being pregnant or made someone pregnant | -0.288 | .390 | 0.349 | .750 | 1.611 | .461 |
| Gender * Working to help the family | -0.079 | .378 | 0.441 | .924 | 1.936 | .834 |
| Gender * Not enough money to pay for school fees | -0.116 | .446 | 0.371 | .890 | 2.135 | .795 |
| Gender * Had to help with looking after the house and siblings | -0.049 | .462 | 0.385 | .952 | 2.355 | .916 |
| Gender * Problems with your school work, teachers or the learners | -0.329 | .422 | 0.315 | .720 | 1.647 | .437 |
| Gender * The school was too far | 0.064 | .485 | 0.412 | 1.066 | 2.759 | .895 |
| Gender * Other | -0.326 | .409 | 0.323 | .721 | 1.610 | .425 |
| Being pregnant or made someone pregnant *Urban (rural ref) | -0.613* | .240 | 0.338 | .542 | 0.867 | .011 |
| Being pregnant or made someone pregnant * Peri-urban | -0.246 | .262 | 0.468 | .782 | 1.308 | .349 |
| Not enough money to pay for school fees * Urban (rural ref) | 1.449 | .734 | 1.011 | 4.259 | 17.942 | .048 |
| Not enough money to pay for school fees *Peri-urban | -0.221 | .747 | 0.185 | .801 | 3.464 | .767 |
| Other * Kwazulu Natal (ref Gauteng) | 0.595 | .336 | 0.939 | 1.814 | 3.505 | .077 |
| Other * Mpumalanga | 1.325* | .360 | 1.858 | 3.761 | 7.616 | .000 |
| Other * Western Cape | 0.353 | .341 | 0.729 | 1.423 | 2.778 | .302 |
| Gender * Kwazulu Natal (ref Gauteng) | 0.125 | .225 | 0.728 | 1.133 | 1.761 | .580 |
| Gender * Mpumalanga | -0.996* | .255 | 0.224 | .369 | 0.609 | .000 |
| Gender * Western Cape | 0.532* | .231 | 1.083 | 1.703 | 2.676 | .021 |
| Gender * Urban (rural ref) | 0.059 | .212 | 0.701 | 1.060 | 1.605 | .781 |
| Gender * Peri-urban | -0.219 | .231 | 0.511 | .803 | 1.263 | .342 |
| Gender * Not enough money to pay for school fees *Urban (rural ref) | -1.098* | .511 | 0.123 | .334 | 0.907 | .032 |
| Gender * Not enough money to pay for school fees * Peri-urban | 0.283 | .500 | 0.498 | 1.328 | 3.541 | .571 |
| Constant | 1.401 | .569 |  | 4.057 |  | .014 |

^Multivariate logistic regression used to generate p-values, *p<.05 indicates significance, Beta (B), Standard error (S.E)^

Figure 1 Respondent Driven Sampling for Out-of-School Youth-A Graphical Illustration of Two Sampling Phases


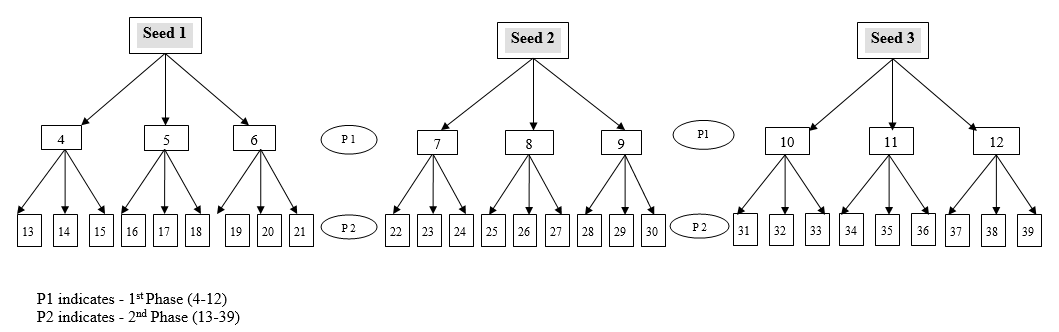


Note: From “Health Risk Behaviours, life skills and socio-economic status survey of Out-of-School Youth in South Africa: An investigation into sexual and other behaviours that place the next generation at risk” by Reddy et al.2011, Cape Town: South African Medical Research Council
